# Development and validation of a smartphone-based deep-learning-enabled system to detect middle-ear conditions in otoscopic images

**DOI:** 10.1038/s41746-024-01159-9

**Published:** 2024-06-20

**Authors:** Constance Dubois, David Eigen, François Simon, Vincent Couloigner, Michael Gormish, Martin Chalumeau, Laurent Schmoll, Jérémie F. Cohen

**Affiliations:** 1 Inserm UMR1153 (CRESS), Université Paris Cité, Paris, France; 2 Clarifai, New York, NY, USA; 3 Department of Pediatric Otolaryngology, Necker-Enfants malades Hospital, APHP, Université Paris Cité, Paris, France; 4 Department of General Pediatrics and Pediatric Infectious Diseases, Necker-Enfants malades Hospital, APHP, Université Paris Cité, Paris, France; 5 NeMo Health, Strasbourg, France

**Keywords:** Diagnosis, Medical imaging

## Abstract

Middle-ear conditions are common causes of primary care visits, hearing impairment, and inappropriate antibiotic use. Deep learning (DL) may assist clinicians in interpreting otoscopic images. This study included patients over 5 years old from an ambulatory ENT practice in Strasbourg, France, between 2013 and 2020. Digital otoscopic images were obtained using a smartphone-attached otoscope (Smart Scope, Karl Storz, Germany) and labeled by a senior ENT specialist across 11 diagnostic classes (reference standard). An Inception-v2 DL model was trained using 41,664 otoscopic images, and its diagnostic accuracy was evaluated by calculating class-specific estimates of sensitivity and specificity. The model was then incorporated into a smartphone app called i-Nside. The DL model was evaluated on a validation set of 3,962 images and a held-out test set comprising 326 images. On the validation set, all class-specific estimates of sensitivity and specificity exceeded 98%. On the test set, the DL model achieved a sensitivity of 99.0% (95% confidence interval: 94.5–100) and a specificity of 95.2% (91.5–97.6) for the binary classification of normal vs. abnormal images; wax plugs were detected with a sensitivity of 100% (94.6–100) and specificity of 97.7% (95.0–99.1); other class-specific estimates of sensitivity and specificity ranged from 33.3% to 92.3% and 96.0% to 100%, respectively. We present an end-to-end DL-enabled system able to achieve expert-level diagnostic accuracy for identifying normal tympanic aspects and wax plugs within digital otoscopic images. However, the system’s performance varied for other middle-ear conditions. Further prospective validation is necessary before wider clinical deployment.

## Introduction

An accurate diagnosis of middle-ear conditions has the potential to reduce both hearing impairment and antimicrobial resistance^1,2^. In low- and middle-income countries, at least 50% of otitis media (OM) cases will lead to hearing impairment^2^. If left untreated, middle-ear conditions can also lead to a wide range of complications, such as balance problems, meningitis, or brain abscess. In high-income countries, middle-ear diseases such as acute otitis media (AOM) are a common cause of children presenting to healthcare providers^3,4^, often leading to inappropriate antibiotic use and driving the emergence of antimicrobial resistance.

Diagnosis of middle-ear conditions relies on otoscopy and functional testing of the eardrum. Initial diagnosis and triage of patients are usually made by primary care providers. However, otoscopy remains challenging for many physicians^5^, and may be inaccessible in remote and resource-limited settings such as nursing homes for the elderly, rural or poor areas, and developing countries^6^. New diagnostic tools for middle-ear conditions are needed to increase accuracy and reproducibility, reduce barriers to accessing healthcare, and improve patient outcomes through early diagnosis, appropriate referral, and treatment.

Deep learning (DL) has shown promise in accurately interpreting images in several areas of clinical medicine^7,8^. DL allows for automatically extracting the most predictive features needed for classification through multiple layers of representation directly from raw images^7,9,10^. Non-experts healthcare providers could use DL-assisted otoscopy to achieve an accurate diagnosis, thereby improving the detection of patients with middle-ear abnormalities that require primary-care level interventions (e.g., antibiotics) or referral to ear-nose-throat (ENT) specialists. Several teams have developed DL-assisted systems for diagnosing middle-ear conditions, with global accuracy usually above 90% (Supplementary Table 1)^11–22^. However, to our knowledge, there is no end-to-end, user-friendly, and highly accurate DL-enabled smartphone-based diagnostic system that is sufficiently validated for clinical practice.

In this study, we describe the development and validation of an end-to-end diagnostic system that combines a smartphone-attached digital otoscope, a DL-enabled model, and a smartphone app for detecting middle-ear conditions.

## Results

### Datasets

**Fig. 1:**
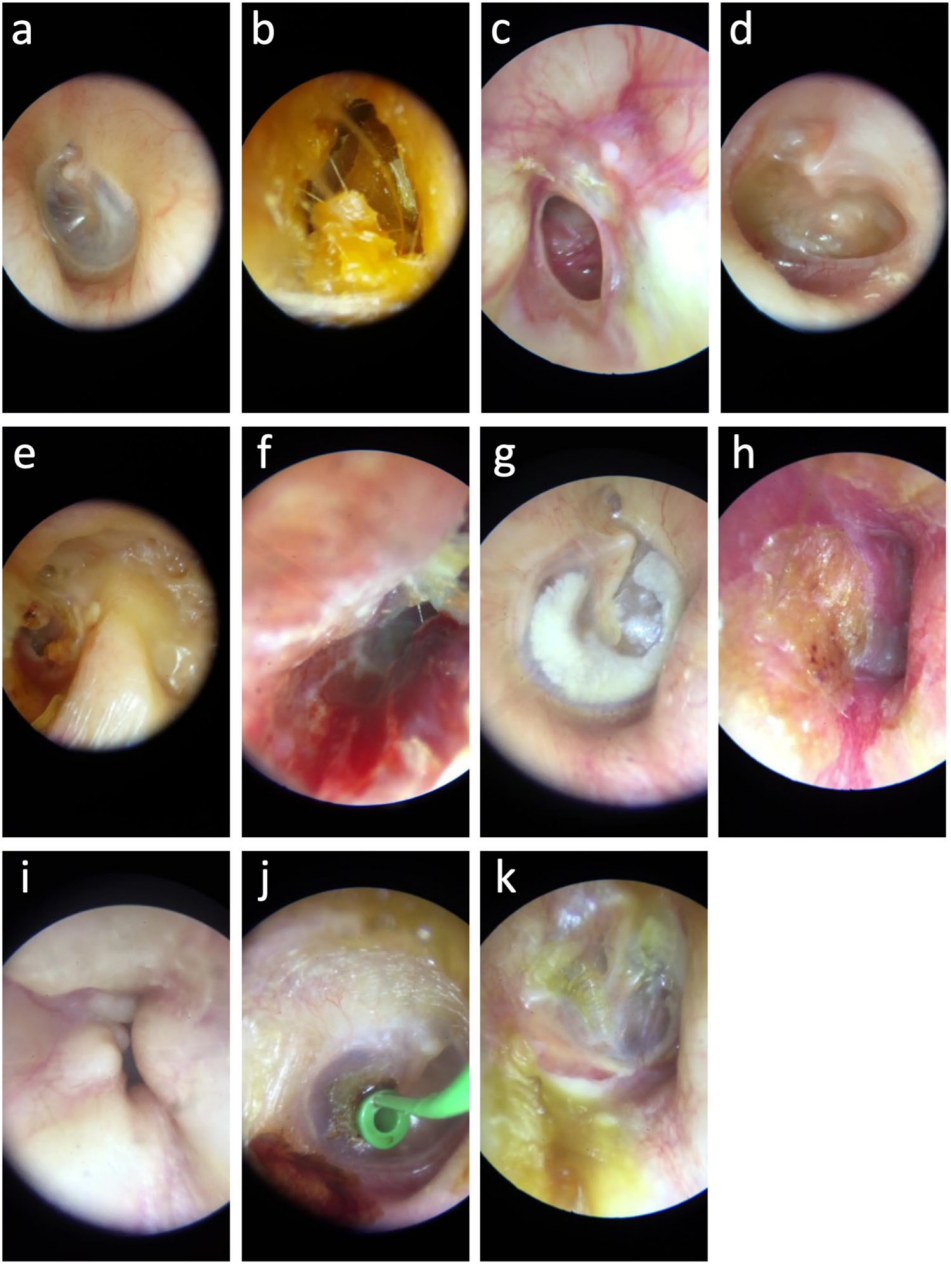
Examples of digital otoscopic images from the dataset. **a** Normal, **b** wax plug, **c** eardrum perforation, **d** otitis media with effusion, **e** cavity after cholesteatoma removal, **f** otitis externa, **g** tympanosclerosis, **h** acute otitis media, **i** external auditory canal osteoma, **j** foreign body, **k** tympanic graft.

A total of 98,137 digital otoscopic images were available for model development. Class balancing was applied to the training and validation sets by capping the number of images used for each class at a maximum of 6000 (uniformly sampled); this helped reduce class imbalance, but rare conditions remained less represented (range: 4% to 13%, Table 1). After class balancing, the final database used for developing the algorithm consisted of 45,606 images that were randomly partitioned into training (41,664 images) and validation (3962 images) sets. The held-out test set included 326 images. Supplementary Figure 1 presents a flowchart of the different datasets. Distributions of the diagnoses in the training, validation, and test sets are shown in Table 1. Examples of images for the 11 categories are presented in Fig. 1. Among the 45,606 images included in DL training and validation, an analysis identified 20,320 images with a high level of similarity (i.e., duplicates), and we estimated that 25,286 otoscopic images used for model development were unique (Supplementary Table 2).

**Table 1:** Distribution of the 11 diagnostic classes in the original dataset, training set, and test set

Diagnosis	Number of images in the original dataset (%)	Number of images in the training set (%)	Number of images in the validation set (%)	Number of images in the test set (%)
Normal	31,706 (32)	5470 (13)	530 (13)	99 (30)
Wax plug	23,583 (24)	5471 (13)	529 (13)	67 (21)
Eardrum perforation	13,652 (14)	5474 (13)	526 (13)	51 (16)
Otitis media with effusion	6958 (7)	5510 (13)	489 (12)	12 (4)
Cavity after cholesteatoma removal	6610 (7)	5469 (13)	531 (13)	26 (8)
Otitis externa	3112 (3)	2831 (7)	280 (7)	10 (3)
Tympanosclerosis	3093 (3)	2826 (7)	267 (7)	14 (4)
Acute otitis media	2949 (3)	2691 (6)	258 (7)	12 (4)
Osteoma	2658 (3)	2456 (6)	202 (5)	14 (4)
Foreign body	2080 (2)	1904 (5)	176 (4)	12 (4)
Tympanic graft	1736 (2)	1562 (4)	174 (4)	9 (3)
Total	98,137 (100)	41,664 (100)	3962 (100)	326 (100)

### Diagnostic accuracy of the deep-learning algorithm on the validation set

**Fig. 2:**
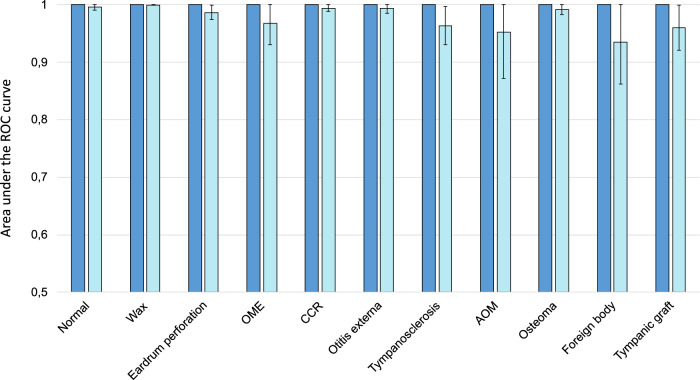
Class-specific estimates of area under the receiver operating characteristic curve (AUROC) with their 95% confidence intervals. Dark blue bars: validation set (*N* = 3,962); Light blue bars: Test set (*N* = 326). All AUROCs on the validation set estimated at 1.00 (1.00–1.00).

**Fig. 3:**
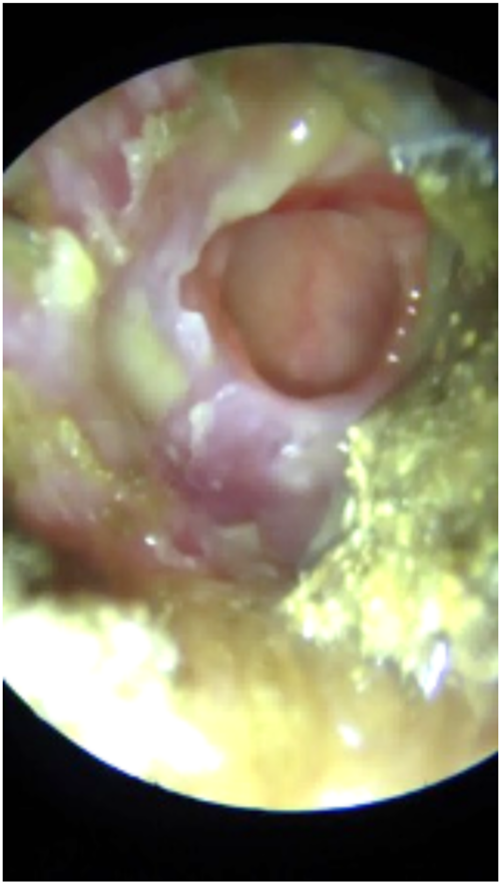
Image of eardrum perforation misclassified by the deep-learning algorithm as otitis externa. On this image, both eardrum perforation and otitis externa are present.

The contingency table reflecting the assessment of the DL algorithm on the validation set is presented in Table 2; Table 3A summarizes corresponding class-specific estimates of sensitivity, specificity, and AUROC; Fig. 2 displays class-specific estimates of AUROC with their 95% confidence intervals; Supplementary Figure 2A presents class-specific ROC curves. For the binary classification of normal vs. abnormal images, the model achieved a sensitivity of 99.1% (97.8–99.7), a specificity of 100% (99.8–100) and an AUROC of 1.00 (1.00–1.00). Class-specific estimates of sensitivity ranged from 98.1% (for eardrum perforation) to 100% (for wax plug); class-specific estimates of specificity ranged from 99.8% (for otitis externa) to 100% (for eardrum perforation, AOM, osteoma, foreign body, and tympanic graft). All class-specific AUROCs were of 1.00 (1.00–1.00) in the validation set. The DL model misdiagnosed nine eardrum perforation images as otitis externa and one as wax plug; in all instances, both eardrum perforation and otitis externa were present (see example in Fig. 3).

**Table 2:** Contingency table for assessment of the deep-learning algorithm across 11 diagnostic classes on the validation set (*N* = 3,962)

	“Ground truth” label											
Deep-learning prediction	Normal	Wax plug	Eardrum perforation	OME	CCR	Otitis externa	Tympanosclerosis	AOM	Osteoma	Foreign body	Tympanic graft	TOTAL
Normal	525	0	0	1	0	0	0	0	0	0	0	526
Wax plug	1	529	1	0	1	0	0	0	0	0	0	532
Eardrum perforation	0	0	516	0	0	0	0	0	0	0	0	516
OME	2	0	0	488	0	0	0	0	0	0	0	490
CCR	0	0	0	0	530	0	0	0	0	0	1	531
Otitis externa	0	0	9	0	0	280	0	0	0	0	0	289
Tympanosclerosis	2	0	0	0	0	0	267	0	0	0	0	269
AOM	0	0	0	0	0	0	0	258	0	0	0	258
Osteoma	0	0	0	0	0	0	0	0	202	0	0	202
Foreign body	0	0	0	0	0	0	0	0	0	176	0	176
Tympanic graft	0	0	0	0	0	0	0	0	0	0	173	173
TOTAL	530	529	526	489	531	280	267	258	202	176	174	3962

*AOM* acute otitis media, *CCR* cavity after cholesteatoma removal, *OME* otitis media with effusion.

**Table 3:** Class-specific estimates of diagnostic accuracy in the validation and test sets

Diagnosis	Sensitivity, % (95%CI)	Specificity, % (95%CI)	AUROC (95%CI)
**3A. Validation set (*N*** **=3,962)**			
Normal	99.1 (97.8–99.7)	100 (99.8–100)	1.00 (1.00–1.00)
Wax plug	100 (99.3–100)	99.9 (99.7–100)	1.00 (1.00–1.00)
Eardrum perforation	98.1 (96.5–99.1)	100 (99.9–100)	1.00 (1.00–1.00)
Otitis media with effusion	99.8 (98.9–100)	99.9 (99.8–100)	1.00 (1.00–1.00)
Cavity after cholesteatoma removal	99.8 (99.0–100)	100 (99.8–100)	1.00 (1.00–1.00)
Otitis externa	100 (98.7–100)	99.8 (99.5–99.9)	1.00 (1.00–1.00)
Tympanosclerosis	100 (98.6–100)	99.9 (99.8–100)	1.00 (1.00–1.00)
Acute otitis media	100 (98.6–100)	100 (99.9–100)	1.00 (1.00–1.00)
Osteoma	100 (98.2–100)	100 (99.9–100)	1.00 (1.00–1.00)
Foreign body	100 (97.9–100)	100 (99.9–100)	1.00 (1.00–1.00)
Tympanic graft	99.4 (96.8–100)	100 (99.9–100)	1.00 (1.00–1.00)
**3B. Test set (*N*** **= 326)**			
Normal	99.0 (94.5–100)	95.2 (91.5–97.6)	1.00 (0.99–1.00)
Wax plug	100 (94.6–100)	97.7 (95.0–99.1)	1.00 (1.00–1.00)
Eardrum perforation	90.2 (78.6–96.7)	96.0 (93.0–98.0)	0.99 (0.97–1.00)
Otitis media with effusion	58.3 (27.7–84.8)	98.1 (95.9–99.3)	0.97 (0.93–1.00)
Cavity after cholesteatoma removal	92.3 (74.9–99.1)	98.3 (96.2–99.5)	0.99 (0.99–1.00)
Otitis externa	70.0 (34.8–93.3)	99.4 (97.7–99.9)	0.99 (0.98–1.00)
Tympanosclerosis	50.0 (23.0–77.0)	99.0 (97.2–99.8)	0.96 (0.93–1.00)
Acute otitis media	58.3 (27.7–84.8)	100 (98.8–100)	0.95 (0.87 –1.00)
Osteoma	57.1 (28.9–82.3)	100 (98.8–100)	0.99 (0.98 –1.00)
Foreign body	33.3 (9.9–65.1)	99.7 (98.2–100)	0.94 (0.86 –1.00)
Tympanic graft	33.3 (7.5–70.1)	99.1 (97.3–99.8)	0.96 (0.92 –1.00)

*AUROC* area under receiver operating characteristic curve.

### Diagnostic accuracy of the deep-learning algorithm on the test set and robustness analysis

The contingency table reflecting the assessment of the DL algorithm on the held-out test set is presented in Table 4; Table 3B summarizes corresponding class-specific estimates of sensitivity, specificity, and AUROC; Fig. 2 displays class-specific estimates of AUROC with their 95% confidence intervals; Supplementary Figure 2B presents class-specific ROC curves. For the binary classification of normal vs. abnormal images, sensitivity was 99.0% (94.5–100), specificity 95.2% (91.5–97.6), and AUROC 1.00 (0.99–1.00). Class-specific sensitivity estimates on the test set ranged from 33.3% (for tympanic graft and foreign body) to 100% (for wax plug). Class-specific specificity estimates ranged from 95.2% (for normal) to 100% (for AOM and osteoma). Class-specific AUROC ranged from to 0.94 (for foreign body) to 1.00 (for normal and wax plug).

**Table 4:** Contingency table for assessment of the deep-learning algorithm across 11 diagnostic classes on the test set (*N* = 326)

	“Ground truth” label											
Deep-learning prediction	Normal	Wax plug	Eardrum perforation	OME	CRC	Otitis externa	Tympano- sclerosis	AOM	Osteoma	Foreign body	Tympanic graft	TOTAL
Normal	98	0	1	1	0	0	3	1	1	4	0	**109**
Wax plug	0	67	0	1	1	0	0	0	2	2	0	**73**
Eardrum perforation	1	0	46	2	0	2	0	3	0	1	2	**57**
OME	0	0	0	7	1	0	3	0	1	0	1	**13**
CCR	0	0	2	0	24	0	0	0	0	0	3	**29**
Otitis externa	0	0	0	0	0	7	0	1	1	0	0	**9**
Tympanosclerosis	0	0	1	1	0	0	7	0	0	1	0	**10**
AOM	0	0	0	0	0	0	0	7	0	0	0	**7**
Osteoma	0	0	0	0	0	0	0	0	8	0	0	**8**
Foreign body	0	0	0	0	0	0	1	0	0	4	0	**5**
Tympanic graft	0	0	1	0	0	1	0	0	1	0	3	**6**
Total	**99**	**67**	**51**	**12**	**26**	**10**	**14**	**12**	**14**	**12**	**9**	**326**

*AOM* acute otitis media, *CCR* cavity after cholesteatoma removal, *OME* otitis media with effusion.

Robustness analysis conducted on corrupted test set images revealed a relative accuracy of ≥80% for most “blur,” “brightness” and “digital” corruptions up to level 3 of severity. However, lower relative accuracy was observed in “noise” experiments and levels 4–5 of severity (Supplementary Table 3).

### Model interpretation

**Fig. 4:**
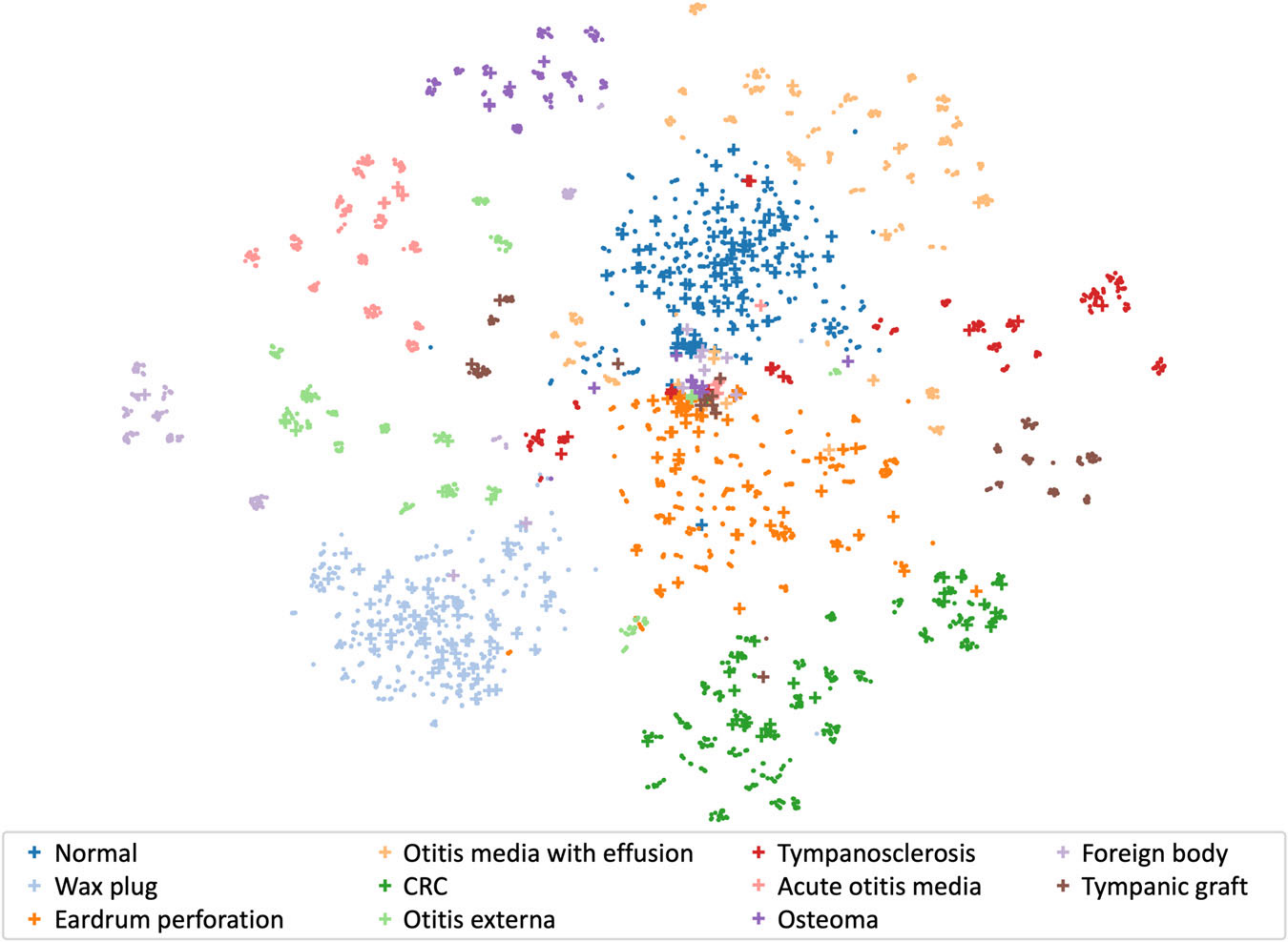
t-Distributed Stochastic Neighbor Embedding (t-SNE) visualization of high-dimensional features extracted by the deep-learning model. Each point represents an otoscopic image (dots: validation set; crosses: test set) and is colored based on its diagnostic class.

The t-SNE plot (Fig. 4) showed how the features extracted by the DL model separated the eleven diagnostic classes into clusters. Overall, most test set points were aggregated in the same cluster as the validation points for their class. Some classes, such as “wax plug,” “normal,” and “acute otitis media” appeared highly aggregated. However, other classes such as “tympanosclerosis,” “foreign body,” and “tympanic graft” were more scattered in the t-SNE space; these three classes also yielded the lowest accuracy estimates.

### Analysis of deep-learning model misclassifications

Among the 326 images in the test set, 48 were misclassified by the DL model (Supplementary Table 4). For 63% (30/48) of misclassified images, the correct diagnosis did not receive the highest prediction but appeared among the three most probable categories according to the DL model. The most frequent errors were: four foreign bodies misclassified as normal, three tympanoscleroses misclassified as normal, three tympanoscleroses misclassified as OMEs, three AOMs misclassified as perforations, and three tympanic grafts misclassified as cavities after cholesteatoma removal. Six otoscopic images were complex to classify because they actually corresponded to multiple diagnoses, including the one suggested by the DL model. In 77% of cases (37/48), blinded reading by a second ENT agreed with the ground truth label; in two cases, blinded reading pointed to potential mistakes in the ground truth labels.

## Discussion

In this large-scale diagnostic accuracy study, we trained and evaluated a DL-enabled algorithm on a labeled dataset of digital otoscopic images. The diagnostic tool achieved high accuracy on the validation set, with all class-specific estimates of sensitivity and specificity exceeding 98%. On the test set, the DL system achieved high accuracy for the binary diagnosis of normal vs. abnormal images and for the identification of wax plugs, but yielded variable performance across other diagnostic categories.

Prior research has explored the potential of using CNNs to improve the diagnosis of middle-ear conditions from otoscopic images^11–22^, with global accuracies ranging from 83.8% to 97.2% (Supplementary Table 1). The present study expands upon this body of evidence using a large and diverse dataset containing 11 diagnostic classes, and further demonstrates that it may be possible to achieve high accuracy with a smartphone-centered system. All DL models fit their training data closer than unseen data, and here the model had lower performances in the test set compared with the validation set. However, we believe the degree of overfitting can be considered modest since all AUROCs on the test set were ≥ 0.94. Our analysis of classification errors allowed us to identify potential areas for model improvement. Tympanic grafts and cavities after cholesteatoma removal appeared as frequent sources of misclassification. These categories may be excluded from the model because they pertain to specific post-operative contexts where clinicians can rely on history-taking to guide their diagnosis. The issue of multiple diagnoses within an image should also be better addressed, for example, by allowing multiple labels in training. Also, more images of AOM and OME should be added to the training set, as these images were often misclassified. We remain confident that wider data collection – multiple investigators acquiring otoscopic frames in multiple locations – should bring back accuracy closer to the 99% estimates found on validation data. Finally, we detected two frames where blinded reading suggested potential errors in ground truth labels, highlighting the limitation of relying on a single expert as the reference standard.

The i-Nside diagnostic system could have several roles in managing patients with suspected middle-ear conditions^23^. In remote areas and resource-limited settings, the DL-enabled system could be used as a triage test for mass screening and targeted referral^24–27^. In primary care, where clinicians may lack expertise in otoscopy, i-Nside could be used as an add-on test to help improve the accurate detection of middle-ear conditions, in line with the principles of “augmented medicine”^28^. For example, Buyn et al. evaluated the benefit of a DL-enabled digital otoscopy model designed to help residents diagnose middle-ear diseases and showed an increase of 7% in accuracy^15^. DL-enabled otoscopy also has potential applications in telemedicine^24,25^: patients could acquire otoscopic images themselves and then send them for DL-assisted interpretation. At the moment, because the test set evaluation only points to high accuracy for distinguishing between normal vs. abnormal images and detecting wax plugs, i-Nside’s primary utility appears to lie in the triage of patients for targeted referrals. An approach to maximize clinical utility could be to reduce the number of output classes by grouping them according to their consequences in terms of patient management, such as “Normal” vs. “Apply home treatment and reevaluate” vs. “Refer to specialist.”

Our DL diagnostic system relies on a single still image of the patient ear as input, while video-otoscopy may enable better assessment. For example, Binol et al. developed a DL automated frame-segmentation, selection, and stitching tool to create enhanced composite images from otoscopy video clips^29–31^. Diagnostic performance was slightly higher when using composite images than a manually selected keyframe (sensitivity of 80% vs. 77%, specificity of 94% vs. 93%, respectively). Other smartphone-enabled innovative avenues to diagnose middle ear conditions exist. For example, several studies have shown that acoustic reflectometry may replace tympanometry for middle ear fluid detection^32,33^. Two studies recorded and analyzed echoes from a device linked to a smartphone and showed promising performance compared with traditional diagnostic tools. However, both systems were evaluated in small proof-of-concept studies with less than 100 patients and deserve independent evaluation. Further improvements might be achieved by combining the signal of such acoustic tests with outputs from DL-enabled otoscopic systems on a smartphone.

Compared with other medical fields where DL-enabled diagnostic systems have been developed – and sometimes FDA-approved^7,34^ – our results seem promising. Most approved artificial intelligence-enabled systems are in radiology and ophthalmology^34^, where large datasets are readily available. For example, a recent external validation study of the Rayvolve® system included 5865 radiographic images from 2549 children and showed high accuracy in detecting fractures, with a sensitivity of 96% and a specificity of 91%^35^. Also, an FDA-cleared and CE-marked DL device to detect diabetic retinopathy in retinal fundus photographs was recently assessed in a prospective multicenter diagnostic study which reported a sensitivity of 95% and a specificity of 85-89%^36^.

Our study has several strengths. First, to our knowledge, we used the largest digital otoscopy dataset to train the model, allowing for a sufficient diversity of images in each class and limiting overfitting. Second, we conducted a validation on a held-out test set to scrutinize whether model performance remained high when applied to new images independent from the training and validation sets; this allowed us to be cautious about our findings and identify the need for further external validation studies. Third, to our knowledge, no other end-to-end diagnostic system using state-of-the-art CNN methods with external validation is available. Cavalcanti et al. developed a smartphone-based otoscope with an accompanying app. However, their study included only 69 patients, and the algorithm relied on an out-of-date multilayer perceptron model that may contribute to the low accuracy of the tool (79.6%)^19^. Myburgh et al. also presented a smartphone- and cloud-based automated OM diagnostic system, but their models used a decision tree and a feedforward one-hidden-layer neural network and had only 389 otoscopic images available for training and testing^20^. These techniques are notoriously less performant than CNNs for image recognition. Chen et al. developed a smartphone-based diagnostic system for ten diagnostic classes, trained on 2161 images^22^. However, they did not evaluate their model on a held-out test set for external validation; they only relied on random-split internal validation, which may lead to over-optimistic performance measures. We found only one study evaluating a CNN tool for detecting middle-ear conditions that included a user-friendly web-based interface^11^. Khan et al. reported a global accuracy of 95.0% for their DL model trained on 2484 images. The main concern is that their algorithm had only three diagnostic classes, whereas our system offers the possibility to diagnose 10 different middle-ear conditions. Our system combines an end-to-end ready-to-use diagnostic solution comprising a smartphone-attached otoscope, a CNN model trained on a large dataset, and a readily available smartphone application.

Our study also has limitations. First, we retrospectively analyzed digital otoscopic images captured during routine care, and thus we lacked important patient characteristics such as age, sex, ethnicity, and presenting signs and symptoms. Furthermore, because of the retrospective nature of the analysis, determining the exact number of unique participants was not practicable. Second, for each image, the final diagnosis was established by a single ENT specialist as per routine care, and tympanometry was performed at the discretion of the clinician in charge. Because of inter-rater variability in otoscopic image interpretation, consensus among several independent ENT experts with systematic tympanometry would have been preferable. Other authors have used more robust reference standards for diagnosing middle-ear conditions. For example, Crowson et al. have relied on findings from the operating room with systematic myringotomy to establish the presence or absence of AOM^16^. However, our analysis of model misclassifications revealed the relatively high reliability of our reference standard, with only 2/48 (4%) of frames pointing to potential mistakes in ground truth labels. Third, all our digital otoscopic images were taken with a single high-performance otoscope, and we anticipate the DL algorithm may achieve lower accuracy with other digital otoscopes, notably those of lower quality. Further research is needed to evaluate whether our DL-based model can be used with other devices. Fourth, because a single operator took all the digital otoscopic images, they may be too homogenous in terms of framing and anatomic perspective. It remains possible that, even with the same otoscope, the DL model would achieve lower performance in the hands of other clinicians. Fifth, the study was conducted in a single center in France, and the case mix may differ in other settings. Of note, the study included children, but only after 5 years of age, while obtaining and interpreting otoscopic images is particularly challenging in young children^37^. Better generalizability might be achieved by increasing the diversity of training data. We call for initiatives to share otoscopic images through open-access platforms to gather large, high-quality, prospectively collected, annotated datasets, as done in other fields exploring medical applications of DL^38^. Sixth, we acknowledge that, although Inception-v2 was an important milestone in the development of CNN classifiers, it may be outperformed by more recent models, including other CNN architectures or Vision Transformers^39,40^. In a post-hoc analysis, we trained other computer vision models (i.e., Inception-v3, ResNet-50, ResNet-101, and ViT_Large) and found limited incremental performance compared with our model (Supplementary Table 5). Seventh, we conducted a validation step on held-out frames, but the test set included only 326 images, and several diagnostic classes, such as AOM and otitis externa, were underrepresented. Finally, our DL model uses solely otoscopic images as input, while combining images with clinical data and patient history in an ensemble model may improve predictive accuracy.

In conclusion, we report the development and validation of a smartphone-based DL-enabled system to detect middle-ear conditions in otoscopic images. Our results on the validation set showed expert-level performance, but the test set revealed sufficient diagnostic accuracy only for distinguishing between normal and abnormal otoscopic images, and for detecting wax plugs. Prospective external validation is required before considering widespread clinical deployment, notably for several diagnostic categories for which test data were scarce.

## Methods

This study is reported according to the CLAIM checklist^41^ (Supplementary Table 6), a specific reporting guideline for AI-centered diagnostic accuracy studies.

### Development of the deep-learning algorithm

For algorithm development, we recruited all consecutive patients over 5 years old who presented to one private ENT practice in Strasbourg, France, from May 2013 to December 2017. For further performance assessment, a “held-out” test set was built with unique images collected in the same conditions over 2018-2020, without any overlap with the training and validation sets, ensuring that images from the same ear or different ear from the same person were not included across the validation and test datasets. All participants were evaluated by a single ENT specialist (L.S.) with more than 20 years of clinical experience. For each patient, both ears underwent otoscopic examination.

**Fig. 5:**
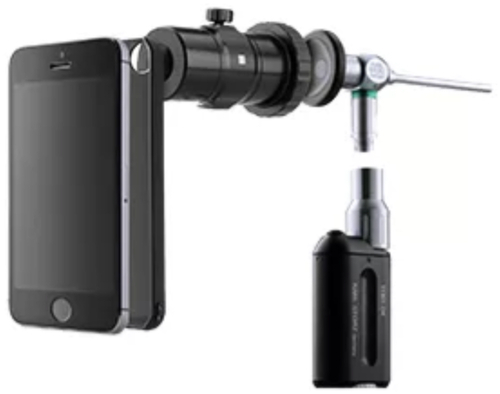
The Smart Scope system. The Smart Scope serves as a coupling system between an endoscope and a smartphone (clinical demonstration video available at i-nside.com).

Digital otoscopic images (and videos) were taken using the Smart Scope (Karl Storz, Tuttlingen, Germany; Fig. 5). The Smart Scope is a universal adapter that enables coupling a handheld endoscope to a smartphone. The adapter is connected to the endoscope via quick-release coupling, and the other end of the adapter is attached to the smartphone camera through a specific smartphone case. The Smart Scope was specifically designed for mobile use, and attachment to the smartphone allows image capture and storage without any external monitor. For this study, the Smart Scope was used with a rigid 4-mm diameter 0-degree surgical endoscope (Hopkins system, Karl Storz, Tuttlingen, Germany), together with a wireless LED light source (11301DF, Karl Storz, Tuttlingen, Germany), and an iPhone 6 or 7 (Apple Inc., USA). As part of routine care, all digital otoscopic images were saved in JPEG format on L.S.’s computer, with an initial 1920 by 1080 pixels resolution.

The ENT’s final diagnosis was used as the reference standard. As per routine care, final diagnoses were established during consultation time by combining demographic information, medical history, signs and symptoms, visual information from otoscopy, and tympanometry (when deemed necessary). Routine otoscopic criteria for each diagnostic class are detailed in Supplementary Table 7. Each anonymized image was labeled with the diagnosis established at the time of examination by the ENT in charge across 11 diagnostic classes: normal, wax plug, eardrum perforation, otitis media with effusion (OME), cavity after cholesteatoma removal, otitis externa, tympanosclerosis, AOM, external auditory canal (EAC) osteoma, EAC foreign body, and tympanic graft. Image labels were saved by locating the JPEG images in 11 predefined folders.

Otoscopic images were included in the dataset if the entire tympanic membrane was exposed and quality (including focus and luminosity) was sufficient to establish a definitive diagnosis. Images were excluded if they were out-of-focus, too dark, or blurry. Class balancing was used to ensure each diagnosis had approximately equal representation in the development set. The obtained dataset was randomly split into training and validation sets. Several data augmentation methods were used for the training set, including flips, translations, and random rotation. Input images were downsized to 455 by 256 pixels. Then, to remove the black circular peripheral region present in otoscopic images, each image was center-cropped and resized to 256 by 256 pixels.

Our use of DL encompasses the process of training a deep neural network to perform a classification task using a set of images for which the final diagnosis is already known (i.e., the training set). For each image, the classification determined by the neural network is compared to the final “ground truth” label, and parameters of the network are iteratively tweaked to decrease classification error rate. As this process is repeated for every image in the training set, the network “learns” how to accurately classify images. This allows obtaining model parameters which are then used to make diagnostic predictions on new, unseen images. The DL algorithm used in this study is a convolutional neural network (CNN) that applies computer vision functions to hierarchically extract patterns and features in input images^10,42,43^.

The specific CNN used in this work was based on the Inception-v2 architecture^44^. Transfer learning was used to optimize the training phase, and initial weights for the CNN model were obtained from a baseline DL model pre-trained on the ImageNet database^42^. The DL model comprises multiple stacked convolution and pooling layers with multiple branches, followed by an 11-class *Softmax* activation function. Our final DL model had approximately 4.9 million parameters. The DL model generates an output “score” between 0 and 1 for each diagnostic class. Then, the set of scores are normalized and interpreted as the probability of that condition being present in the image according to the model. DL modeling was implemented in Python by using TensorFlow libraries.

### Evaluation of the deep-learning algorithm

For performance assessment, the algorithm was evaluated on a split-sampling validation set made of 3,962 images and then a held-out test set comprising 326 unique images without overlap with the training and validation sets. For each image, the diagnosis with the highest DL-generated prediction was compared to the reference standard (i.e., “top-1” validation). This allowed drawing contingency tables across the 11 diagnostic classes. Sensitivity, specificity, area under the receiver operating curve (AUROC) and their 95% “exact” binomial confidence intervals were computed for each diagnostic class in the validation and test sets. Diagnostic accuracy analyses were conducted using R version 4.2.1 (R Foundation, Vienna). There was no formal sample size calculation for this study.

To further explore the generalizability of the model, we investigated its robustness following Hendrycks’ method^45^. We used 12 image corruption processes at five levels of severity and estimated the model’s accuracy relative to that obtained on uncorrupted images. We excluded corruption processes mimicking weather-like disturbances (snow, frost, fog) since they are unlikely to occur during otoscopy.

We used the t-distributed stochastic neighbor embedding (t-SNE) visualization method to represent high-dimensional features learned by the DL model. The t-SNE method preserves pairwise distances between data points in a 2-dimensional space (i.e., images with similar features cluster together)^46^. We showed a t-SNE visualization for the penultimate activation layer, computed over validation and test sets.

DL misclassifications in the test set were systematically analyzed. First, we assessed the proportion of cases where the correct diagnosis appeared in the top-3 of model predictions. Second, all images were reviewed by a second ENT specialist (F.S.) blinded to ground truth labels, DL predictions, and clinical data, and we measured the proportion of cases where this blinded reading was aligned with ground truth labels.

### Smartphone app graphical user interface

**Fig. 6:**
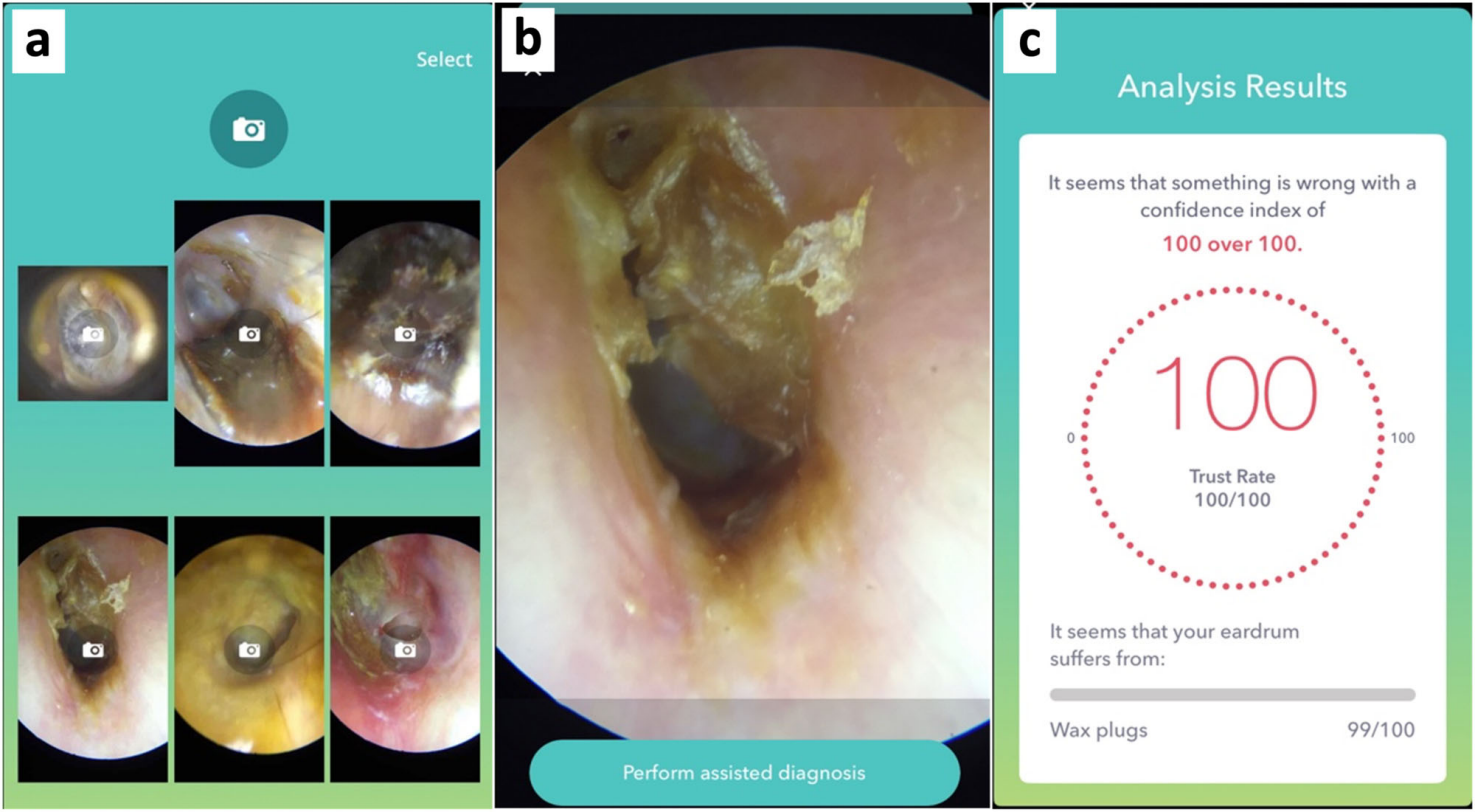
i-Nside smartphone app captions. **a** Home page. **b** Loaded image. **c** Result page.

An easy-to-use smartphone app was developed to allow broad use of the DL model for users with limited technical and statistical knowledge. The i-Nside app was developed by Design and Test Lab, a third-party contractor, using the Xcode interface and several libraries such as Metal, MetalKit, NMSSH, and ClarifaiMobileSDK. Designed to run on iOS, the app is currently at version 1.2 and has a file size of 125 MB. The app was designed to directly capture and analyze images when connected to a Smart Scope digital otoscope. The app also enables the importation of otoscopic images from external sources through the phone’s photo library, accommodating JPEG, PNG, and HEIC formats with an upper size limit of 50 MB (Fig. 6). The app utilizes a local FTP server for uploading photos, ensuring that images are stored locally without being exported to any external server or cloud storage. After an image is selected, the user taps on “Perform assisted diagnosis” to run the DL model (Supplementary Video 1). The app works entirely offline. The model runs in real-time and locally on the smartphone using Clarifai’s Mobile SDK, and the results are displayed in the app within 1 second.

After the image is analyzed, the app displays the most likely diagnosis among the 11 diagnostic classes, and its precise probability. If the “Normal” class receives a model prediction X ≥ 70%, the app displays: “It seems that your eardrum is normal with a confidence index of X%”. If the “Normal” class receives a model probability Y < 70%, the app displays: “It seems that something is wrong with a confidence index of (100-Y)%”; in such cases, the app also returns the most probable diagnostic class, together with its probability. In the case of multiple possible diagnoses in a single image, the app displays all diagnoses assigned a prediction higher than 10%.

### Ethics

Images (and videos) were taken as part of routine care with oral information. Informed written consent was waived by the Institutional Review Board (“Comité d'éthique de la recherche AP-HP Centre,” IRB registration No. 00011928) because of the retrospective nature of the analysis, total deidentification of clinical images, and absence of any other patient information being collected.

### Reporting summary

Further information on research design is available in the Nature Research Reporting Summary linked to this article.

### Supplementary information

Supplementary information

Reporting summary

Supplementary Video

## Data Availability

A sub-sample of the training set, with 100 images per diagnostic class is available upon reasonable request to the authors.

## Code availability

Baseline Inception-v2 architecture can be accessed on the timm repository (huggingface.co/timm). Further code is available upon reasonable request to the authors.
