# Supplementary material for: Development and validation of a smartphone-based deep-learning-enabled system to detect middle-ear conditions in otoscopic images
Source: NPJ Digit Med. 2024 Jun 20;7:162. doi: 10.1038/s41746-024-01159-9 (PMC11189910; doi:10.1038/s41746-024-01159-9)
Supplement: Supplementary file 1 — Supplementary information [file 41746_2024_1159_MOESM1_ESM.pdf]

SUPPLEMENTARY INFORMATION

Development and validation of a smartphone-based deep-learning-enabled system to detect middle-ear conditions in otoscopic images

Constance Dubois, David Eigen, François Simon, Vincent Couloigner,
Michael Gormish, Martin Chalumeau, Laurent Schmoll, Jérémie F. Cohen

Table of contents

Supplementary Table 1. Studies of machine learning and deep learning-assisted systems for diagnosing middle-ear conditions (non-exhaustive review) 2

Supplementary Table 2. Results of image similarity analysis in the database for model development (N=45,606 images) 3

Supplementary Table 3. Robustness analysis 4

Supplementary Table 4. Analysis of DL misclassifications in the test set (N=48/326 images) 5

Supplementary Table 5. Comparison of the i-Nside model with other state-of-the-art deep-learning architectures 7

Supplementary Table 6. CLAIM reporting checklist 8

Supplementary Table 7. Routine otoscopic criteria used for ground truth labeling ... 10

Supplementary Figure 1. Study flowchart 11

Supplementary Figure 2. Receiver operating characteristic (ROC) Curves 12

Supplementary Table 1. Studies of machine learning and deep learning-assisted systems for diagnosing middle-ear conditions (non-exhaustive review)

Author	Year	Journal / Conference name	CNN architecture*	Number of images	Global accuracy *, %	Diagnostic classes	State of development	App / user interface
Basaran	2019	Biomedical Signal Processing and Control	VGG-16	1,692	90.5	7 (normal, AOM, earwax buildup, myringosclerosis, tympanostomy tubes, CSOM, otitis externa)	Internal validation	None
Byun	2021	Journal of Clinical Medicine	ResNet18 + Shuffle Attention Module	2,272	97.2	4 (normal, OME, COM, cholesteatoma)	Internal validation	None
Cai	2021	BMJ open	ResNet50 + focal classifier	6,066	93.4	4 (normal, OME, two stages of CSOM)	Internal validation	None
Cavalcanti	2021	Biomedical Optics Express	Multilayer perceptron**	69	79.6	3 (normal, OME, adhesive OM)	Internal validation	Smartphone app
Cha	2019	EBioMedicine	Ensemble: Inception-V3 + ResNet101	10,544	93.7	6 (normal, OME, attic retraction, tympanic perforation, otitis externa ± myringitis, tumor/earwax)	Internal validation	None
Chen	2022	EClinicalMedicine	MobileNetV2	2,161	97.6	10 (normal, AOM, acute myringitis, CSOM, OME, tympanic membrane perforation, cerumen impaction, ventilation tube, tympanic membrane retraction, and otomycosis)	Internal validation	Smartphone app
Crowson	2021	Pediatrics	ResNet-34	338	83.8	2 (normal, effusion)	Internal validation	None
Khan	2020	Neural Networks	DenseNet	2,484	95.0	3 (normal, COM with TM perforation, OME)	Internal validation	Web-based user interface
Livingstone	2020	Laryngoscope	Google Cloud Vision AutoML	1,458	88.7	14 (normal, AOM, cholesteatoma, earwax buildup, SOM, exostoses, myringitis, myringosclerosis, otomycosis, otitis externa, perforation, TM retraction, tympanostomy tube (present and in position), or tympanostomy tube (extruded))	Internal validation	None
Myburgh	2018	Biomedical Signal Processing and Control	Decision tree and neural network**	389	86.8	5 (normal, wax or foreign body, AOM, OME, CSOM)	Internal validation	Smartphone app
Wu	2021	Laryngoscope	Xception	12,305	90.7	3 (normal, AOM, OME)	External validation (102 images)	None

Abbreviations: AOM, acute otitis media; COM, chronic otitis media; CSOM, chronic suppurative otitis media; OME, otitis media with effusion; SOM, serous otitis media; TM, tympanic membrane.

* for studies evaluating several architectures, we selected the architecture with the best global accuracy.
** not considered deep learning / CNN.

Supplementary Table 2. Results of image similarity analysis in the database for model development (N=45,606 images)

Analysis conducted with dupeGuru software v4.0.3, considering a 95% similarity threshold (default setting) to define duplicates. Among the 45,606 otosocpic images that were included in the analysis, 20,320 had at least one duplicate. We estimated that 25,286 images were unique.

**Parameter**		**Value**
Number of duplicate images in the dataset		
	Normal	2,229
	Wax plug	901
	Eardrum perforation	2,942
	Otitis media with effusion (OME)	3,590
	Cavity after cholesteatoma removal	2,502
	Otitis externa	1,381
	Tympanosclerosis	1,976
	Acute otitis media (AOM)	1,496
	Osteoma	1,490
	Foreign body	854
	Tympanic graft	959
	TOTAL	20,320
**For images with duplicates:**		
Mean number of similar images		1.9
Median number of similar images		1
Q1		1
Q3		2
Minimum number of duplicates per image		1
Maximum number of duplicates per image		59

Supplementary Table 3. Robustness analysis

Data corruptions were applied to test set images at five levels of severity following Hendrycks' method. Model accuracy is reported for each corrupted set ('absolute accuracy') and relative to that obtained on the test set without data corruptions ('relative accuracy').

Corruptions were applied at the final image preprocessing stage before the neural network model rather than at the image input, as this highlights perturbations with the greatest impact.

	Severity = 1		Severity = 2		Severity = 3		Severity = 4		Severity = 5	
Corruption type	Absolute accuracy, %	Relative accuracy*, %	Absolute accuracy, %	Relative accuracy*, %	Absolute accuracy, %	Relative accuracy*, %	Absolute accuracy, %	Relative accuracy*, %	Absolute accuracy, %	Relative accuracy*, %
**Noise**										
Gaussian noise	61.0	71.5	51.2	60.0	42.4	49.7	25.6	30.0	20.4	23.9
Shot noise	57.0	66.8	45.4	53.2	35.1	41.1	20.1	23.6	20.4	23.9
Impulse noise	47.6	55.8	47.9	56.2	37.8	44.3	24.4	28.6	20.4	23.9
**Blur**										
Defocus blur	82.0	96.1	81.1	95.1	71.6	83.9	60.4	70.8	53.4	62.6
Glass blur	84.8	99.4	83.8	98.2	77.1	90.4	75	87.9	72	84.4
Motion blur	83.8	98.2	79.6	93.3	72.3	84.8	65.5	76.8	57.6	67.5
Zoom blur	74.7	87.6	69.5	81.5	64.6	75.7	62.2	72.9	58.8	68.9
**Weather****										
Brightness	84.5	99.1	82.9	97.2	79.6	93.3	69.5	81.5	60.7	71.2
**Digital**										
Contrast	66.8	78.3	56.7	66.5	46.3	54.3	23.2	27.2	20.4	23.9
Elastic transform	82.6	96.8	73.8	86.5	77.1	90.4	70.1	82.2	56.1	65.8
Pixelate	74.7	87.6	73.2	85.8	70.7	82.9	68.3	80.1	67.4	79.0
Jpeg compression	79.9	93.7	74.7	87.6	69.8	81.8	54.6	64.0	35.7	41.9

*Accuracy relative to that obtained with the initial model on the original test set without data corruptions. Initial model accuracy on the original test set without data corruption was 85.3%. ** Other corruption processes mimicking weather-like disturbances (snow, frost, fog) were excluded since they are unlikely to occur during otoscopy.

Supplementary Table 4. Analysis of DL misclassifications in the test set (N=48/326 images)

For each misclassified image, the ground truth label, the DL top-1 diagnosis, and whether the correct label was within its top-3 predictions are presented. All images were also reviewed by an ENT specialist (FS) blinded to ground truth labels, DL predictions, and clinical data, and we scored whether this blinded reading was aligned with ground truth labels.

Ground truth label	DL top-1 prediction	Correct label in DL Top-3 predictions (yes/no)	ENT blinded reading aligned with ground truth label (yes/no)	Commentary and potential explanation for DL error
Normal	Eardrum perforation	Yes	Yes	Tympanic membrane in ear canal might be mistaken as perforation due to the picture being taken shallow in the canal.
Eardrum perforation	Cavity after cholesteatoma removal	Yes	No	Difficult case, atypical image, complex post-operative aspect.
Eardrum perforation	Normal	No	Yes	Difficult case: perforated retraction pocket on a post-operative tympanic membrane.
Eardrum perforation	Cavity after cholesteatoma removal	Yes	Yes	Difficult case: Inflammatory perforation.
Eardrum perforation	Tympanosclerosis	Yes	Yes	Double diagnosis: tympanosclerosis and perforation.
Eardrum perforation	Tympanic graft	Yes	Yes	Difficult case: presence of a deposit of dead skin from perforation.
Otitis media with effusion	Wax plug	No	Yes	No explanation, typical OME.
Otitis media with effusion	Eardrum perforation	Yes	Yes	Typical OME but a small dark zone in the tympanic membrane might have been mistaken for perforation.
Otitis media with effusion	Eardrum perforation	Yes	Yes	Typical OME but a small dark zone in the tympanic membrane might have been mistaken for perforation.
Otitis media with effusion	Normal	Yes	Yes	No explanation, typical OME.
Otitis media with effusion	Tympanosclerosis	No	No	No explanation, typical OME.
Cavity after cholesteatoma removal	Wax plug	Yes	No	Difficult case: Ear canal also filled with wax.
Cavity after cholesteatoma removal	Otitis media with effusion	Yes	No	Difficult post-operative case. Post-operative clinical context of recent graft should help.
Otitis externa	Tympanic graft	Yes	Yes	The clinical context should allow excluding a tympanic graft.
Otitis externa	Eardrum perforation	Yes	Yes	Tympanic membrane in ear canal might be mistaken as perforation due to the picture being taken shallow in the canal.
**Otitis externa**	**Eardrum perforation**	**Yes**	**No**	**Potential error in ground truth label.**
Tympanosclerosis	Otitis media with effusion	No	Yes	Rounded normal tympanic membrane zone might have been mistaken for otitis media with effusion with blister or perforation.
Tympanosclerosis	Foreign body	No	Yes	Typical tympanosclerosis. Wax deposit mistaken as foreign body?
Tympanosclerosis	Otitis media with effusion	Yes	Yes	Typical tympanosclerosis, no explanation.
Tympanosclerosis	Normal	Yes	Yes	Typical tympanosclerosis, no explanation.

Tympanosclerosis	Otitis media with effusion	Yes	Yes	Atypical yellowish tympanosclerosis.
Tympanosclerosis	Normal	No	Yes	Typical tympanosclerosis, no explanation.
Tympanosclerosis	Normal	Yes	Yes	Atypical dotted tympanosclerosis.
Acute otitis media	Eardrum perforation	Yes	No	Difficult case: Blurred picture, small black stain could be a perforation; inflammatory canal.
Acute otitis media	Eardrum perforation	Yes	Yes	Typical acute otitis media. Dark vascular defect mistaken as perforation?
Acute otitis media	Eardrum perforation	No	Yes	Typical acute otitis media, no explanation.
Acute otitis media	Otitis externa	No	Yes	Inflammatory canal with secretions.
Acute otitis media	Normal	No	Yes	Typical acute otitis media, no explanation.
Osteoma	Normal	Yes	Yes	Double diagnosis: Osteoma and normal tympanic membrane.
Osteoma	Otitis media with effusion	No	Yes	Typical osteoma, no explanation.
Osteoma	Otitis externa	Yes	Yes	Tympanic membrane not visible due to osteoma
Osteoma	Tympanic graft	Yes	Yes	Typical osteoma, no explanation
Osteoma	Wax plug	No	Yes	Double diagnosis: There is an osteoma but also some wax deposit and inflammatory ear canal.
Osteoma	Wax plug	No	Yes	Double diagnosis: osteoma and wax.
Foreign body	Normal	No	No	Bead was interpreted as a watery blister by blinded ENT.
**Foreign body**	**Normal**	**No**	**No**	**Potential error in ground truth label.**
Foreign body	Tympanosclerosis	No	No	Triple diagnosis: tympanosclerosis, perforation, and foreign body.
Foreign body	Wax plug	Yes	Yes	Foreign body shape looks like wax, but is too white.
Foreign body	Normal	No	Yes	Yellow foreign body in front of a normal tympanic membrane.
Foreign body	Eardrum perforation	Yes	Yes	Blue tympanostomy tube: round shape might have been mistaken for perforation?
Foreign body	Wax plug	Yes	Yes	Double diagnosis: yellowish tympanostomy tube with a wax plug.
Foreign body	Normal	No	Yes	Transparent tympanostomy tube, no explanation.
Tympanic graft	Eardrum perforation	No	No	Post-operative clinical context of recent graft should help.
Tympanic graft	Otitis media with effusion	Yes	Yes	Post-operative clinical context of recent graft should help.
Tympanic graft	Cavity after cholesteatoma removal	Yes	No	Difficult case: Recent graft, inflammatory, tympanic membrane challenging to identify. DL confused between 2 post-operative aspects.
Tympanic graft	Eardrum perforation	Yes	Yes	Post-operative clinical context of recent graft should help.
Tympanic graft	Cavity after cholesteatoma removal	Yes	Yes	Severe retraction pocket might have been mistaken for cavity after cholesteatoma removal. DL confused between 2 post-operative aspects. Post-operative clinical context of recent graft should help.
Tympanic graft	Cavity after cholesteatoma removal	Yes	Yes	Difficult case: White and thick graft. DL confused between 2 post-operative aspects. Post-operative clinical context of recent graft should help.

Supplementary Table 5. Comparison of the i-Nside model with other state-of-the-art deep-learning architectures

	**Global accuracy, % (95%CI)**	
	Validation set	Test set
Present model (i-Nside)	99.6 (99.3-99.7)	85.3 (81.0-88.9)
Inception-v3*	99.5 (99.2-99.7)	87.8 (83.8-91.1)
ResNet-50*	99.3 (99.0-99.5)	86.0 (81.7-89.6)
ResNet-101*	99.2 (98.9-99.5)	89.3 (85.5-92.5)
ViT_Large**	99.7 (99.4-99.8)	89.3 (85.5-92.5)

Additional models were trained using the PyTorch Image Models (timm). Models pre-trained on *ImageNet1k or **ImageNet22k. All 95% confidence intervals were computed with the 'exact' binomial method.

Supplementary Table 6. CLAIM reporting checklist

Section/Topic	Item	Reported
TITLE OR ABSTRACT		
	1 Identification as a study of AI methodology, specifying the category of technology used (eg, deep learning)	title
ABSTRACT		
	2 Structured summary of study design, methods, results, and conclusions	abstract
INTRODUCTION		
	3 Scientific and clinical background, including the intended use and clinical role of the AI approach	yes
	4 Study objectives and hypotheses	yes
METHODS		
Study Design	5 Prospective or retrospective study	yes
	6 Study goal, such as model creation, exploratory study, feasibility study, noninferiority trial	Model creation
Data	7 Data sources	yes
	8 Eligibility criteria: how, where, and when potentially eligible participants or studies were identified (eg, symptoms, results from previous tests, inclusion in registry, patient-care setting, location, dates)	yes
	9 Data preprocessing steps	yes
	10 Selection of data subsets, if applicable	N/A
	11 Definitions of data elements, with references to common data elements	yes
	12 De-identification methods	yes
	13 How missing data were handled	no
Ground Truth	14 Definition of ground truth reference standard, in sufficient detail to allow replication	yes
	15 Rationale for choosing the reference standard (if alternatives exist)	
	16 Source of ground truth annotations; qualifications and preparation of annotators	yes
	17 Annotation tools	yes
	18 Measurement of inter- and intrarater variability; methods to mitigate variability and/or resolve discrepancies	no
Data Partitions	19 Intended sample size and how it was determined	yes
	20 How data were assigned to partitions; specify proportions	yes
	21 Level at which partitions are disjoint (eg, image, study, patient, institution)	yes

Model	22 Detailed description of model, including inputs, outputs, all intermediate layers and connections	yes
	23 Software libraries, frameworks, and packages	yes
	24 Initialization of model parameters (eg, randomization, transfer learning)	yes
Training	25 Details of training approach, including data augmentation, hyperparameters, number of models trained	yes
	26 Method of selecting the final model	no
	27 Ensembling techniques, if applicable	N/A
Evaluation	28 Metrics of model performance	yes
	29 Statistical measures of significance and uncertainty (eg, confidence intervals)	yes
	30 Robustness or sensitivity analysis	yes
	31 Methods for explainability or interpretability (eg, saliency maps) and how they were validated	no
	32 Validation or testing on external data	yes
RESULTS		
Data	33 Flow of participants or cases, using a diagram to indicate inclusion and exclusion	yes
	34 Demographic and clinical characteristics of cases in each partition	no
Model performance	35 Performance metrics for optimal model(s) on all data partitions	yes
	36 Estimates of diagnostic accuracy and their precision (such as 95% confidence intervals)	yes
	37 Failure analysis of incorrectly classified cases	yes
DISCUSSION		
	38 Study limitations, including potential bias, statistical uncertainty, and generalizability	yes
	39 Implications for practice, including the intended use and/or clinical role	yes
OTHER INFORMATION		
	40 Registration number and name of registry	yes
	41 Where the full study protocol can be accessed	no
	42 Sources of funding and other support; role of funders	yes

Mongan J, Moy L, Kahn CE, Jr. Checklist for Artificial Intelligence in Medical Imaging (CLAIM): A Guide for Authors and Reviewers. Radiol Artif Intell. 2020;2(2):e200029.

Supplementary Table 7. Routine otoscopic criteria used for ground truth labeling

Diagnostic labeling was performed during patient consultation, using demographic information, medical history, signs and symptoms, visual findings from otoscopy (as detailed below), and tympanometry (when deemed necessary).

Diagnostic class	Routine otoscopic criteria (i.e., typical otoscopic aspect)
**Normal**	Intact, grayish, and translucent tympanic membrane, with discernible landmarks such as the umbo and the handle of the malleus.
**Wax plug**	Yellowish or brownish wax in the external auditory canal, which may partially or totally obstruct the view of the tympanic membrane.
**Eardrum perforation**	Hole or rupture in an otherwise intact tympanic membrane, often with a visible rim around the perforation.
**Otitis media with effusion**	Intact tympanic membrane with retrotympanic fluid or air bubbles, without signs of acute infection.
**Cavity after cholesteatoma removal**	Large open cavity within the mastoid region, due to a canal-wall down tympanoplasty procedure; the cavity may contain residual tissue or skin debris.
**Otitis externa**	Inflammation (edema/swelling or erythema/redness) of the external auditory canal, sometimes with discharge such as pus or fluid within the canal.
**Tympanosclerosis**	Presence of white calcifications within the tympanic membrane, typically appearing as opaque or chalky-white patches on the tympanic membrane.
**Acute otitis media**	Bulging and inflammation (edema and erythema) of the tympanic membrane, loss of malleus landmarks, often accompanied by the presence of pus visible behind the tympanic membrane.
**Osteoma**	Bony growth in the external auditory canal, which may partially or totally obstruct the view of the tympanic membrane.
**Foreign body**	Presence of a foreign body in the external auditory canal, which may partially or totally obstruct the view of the tympanic membrane; common examples include beads, insects, cotton swabs, tympanostomy tubes, or small toys.
**Tympanic graft**	Intact post-tympanoplasty tympanic membrane, usually with a retro-tympanic cartilage graft visible as a whitish or translucent patch behind the tympanic membrane.

Supplementary Figure 1. Study flowchart

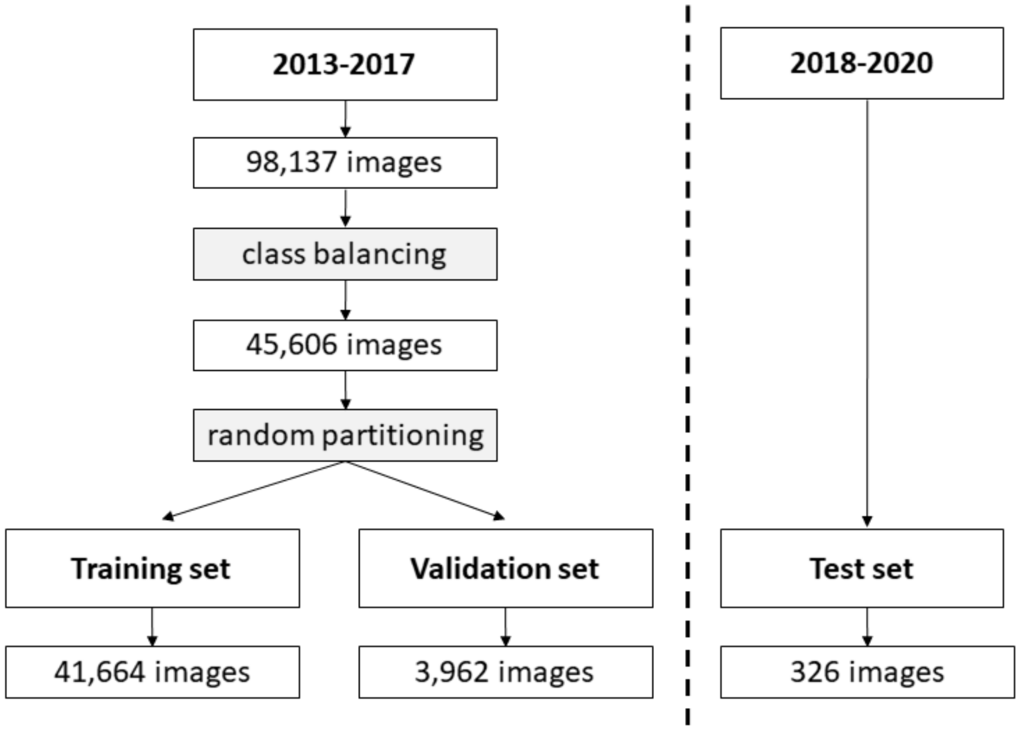

Supplementary Figure 2. Receiver operating characteristic (ROC) Curves

2A-Validation set (N=3,962)

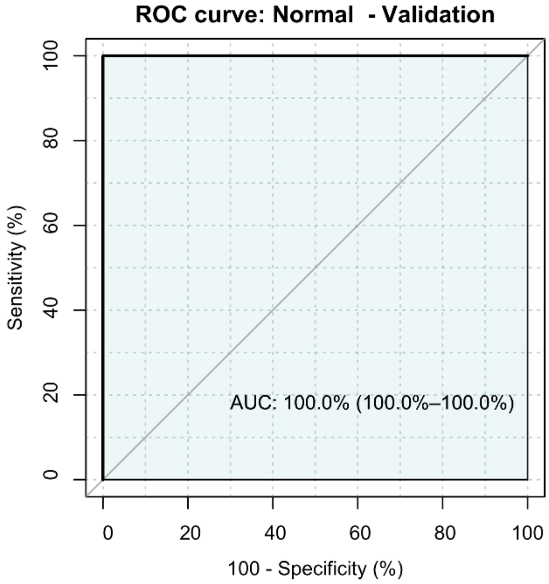

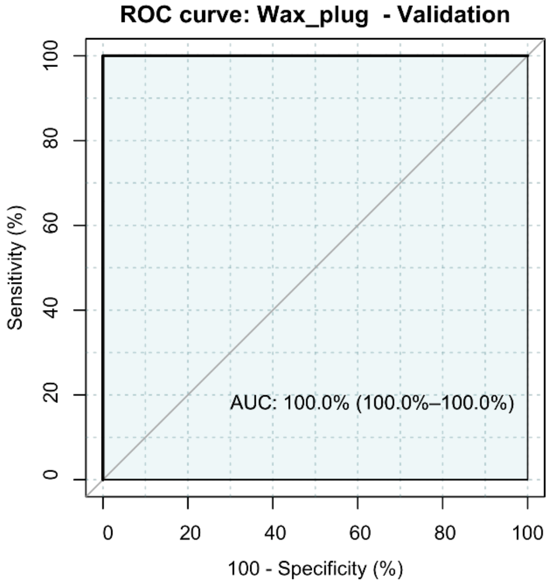

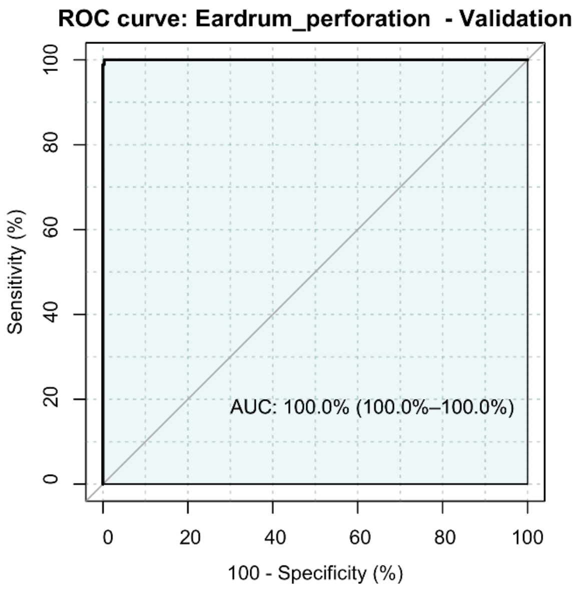
ROC curve: Eardrum_perforation - Validation
Sensitivity (%)
100 - Specificity (%)
AUC: 100.0% (100.0%–100.0%)
0
20
40
60
80
100

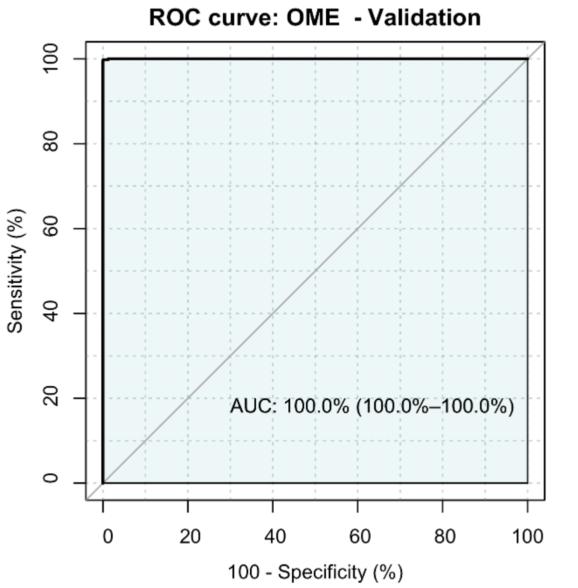
ROC curve: OME - Validation
Sensitivity (%)
100 - Specificity (%)
AUC: 100.0% (100.0%–100.0%)
0
20
40
60
80
100

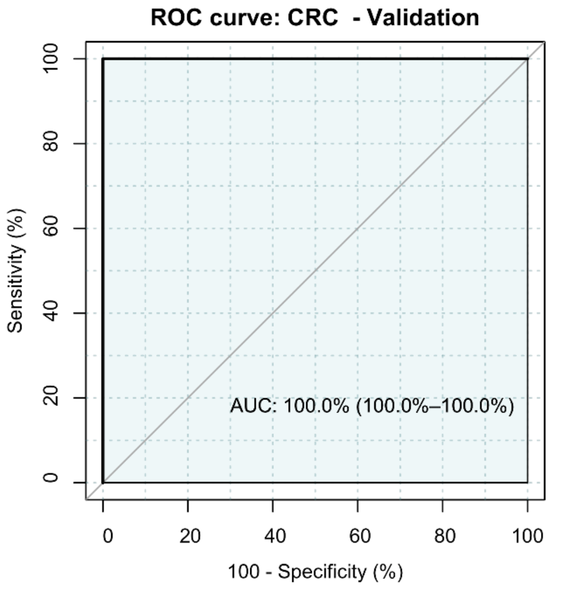
ROC curve: CRC - Validation
Sensitivity (%)
100 - Specificity (%)
AUC: 100.0% (100.0%–100.0%)
0
20
40
60
80
100

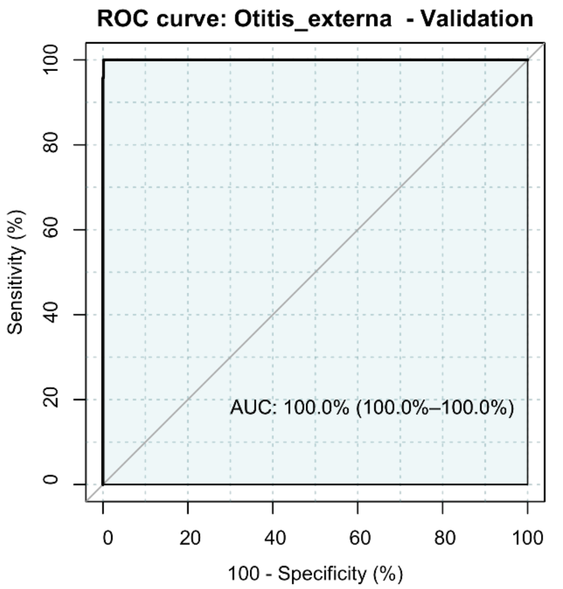
ROC curve: Otitis_externa - Validation
Sensitivity (%)
0
20
40
60
80
100
100 - Specificity (%)
AUC: 100.0% (100.0%–100.0%)

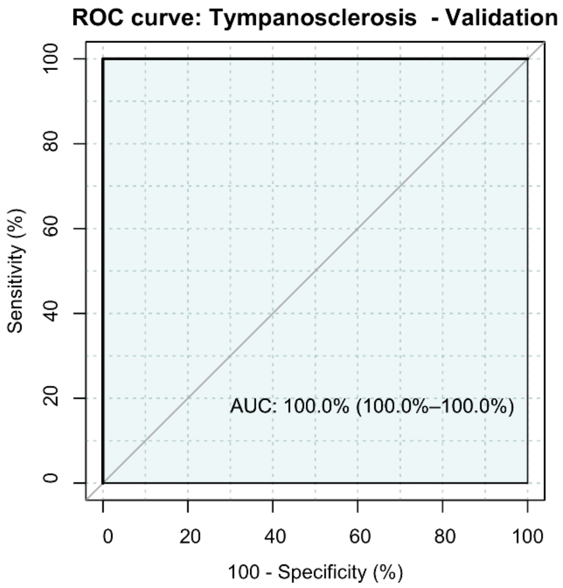
ROC curve: Tympanosclerosis - Validation
Sensitivity (%)
0
20
40
60
80
100
100 - Specificity (%)
AUC: 100.0% (100.0%–100.0%)

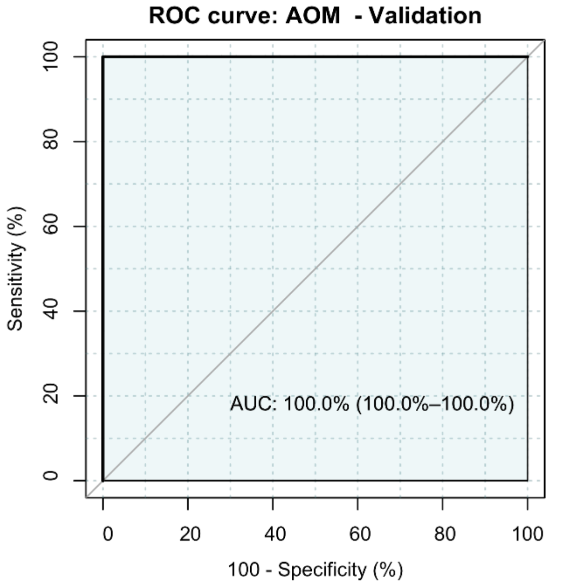
ROC curve: AOM - Validation
Sensitivity (%)
0
20
40
60
80
100
100 - Specificity (%)
AUC: 100.0% (100.0%–100.0%)

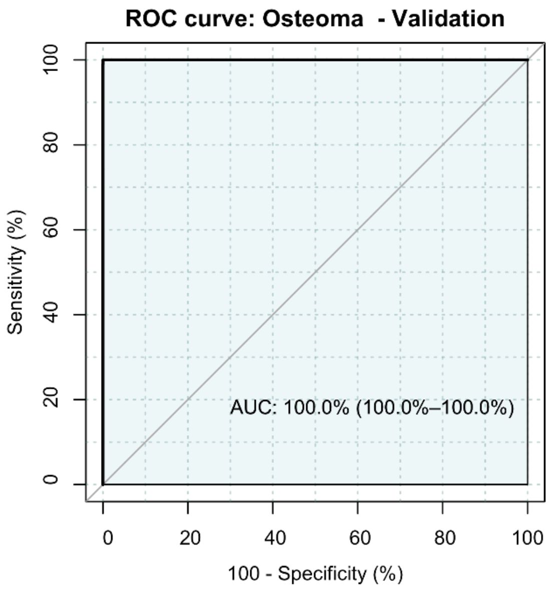
ROC curve: Osteoma - Validation
Sensitivity (%)
100 - Specificity (%)
0
20
40
60
80
100
AUC: 100.0% (100.0%–100.0%)

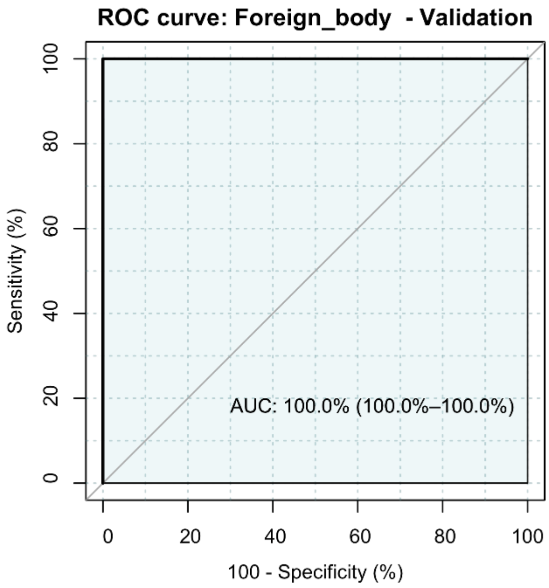
ROC curve: Foreign_body - Validation
Sensitivity (%)
100 - Specificity (%)
0
20
40
60
80
100
AUC: 100.0% (100.0%–100.0%)

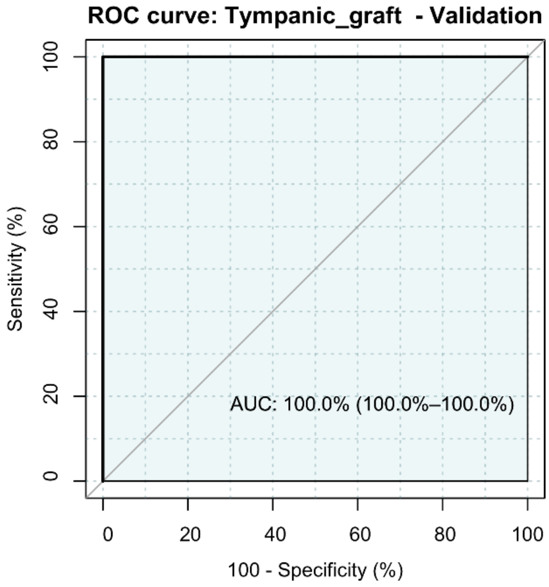
ROC curve: Tympanic_graft - Validation
Sensitivity (%)
100 - Specificity (%)
0
20
40
60
80
100
AUC: 100.0% (100.0%–100.0%)

2B-Test set (N=326)

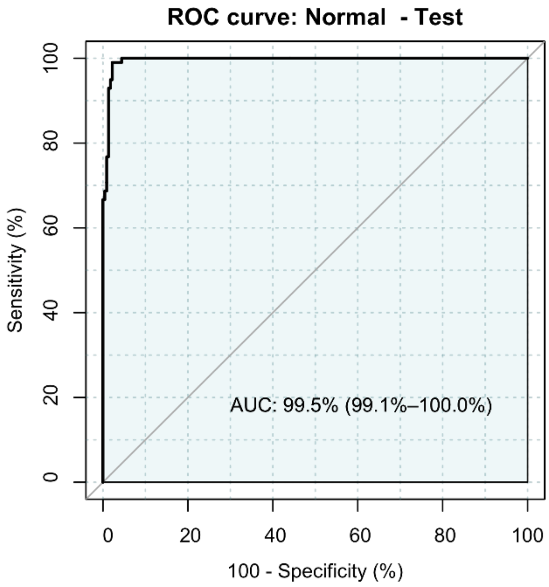

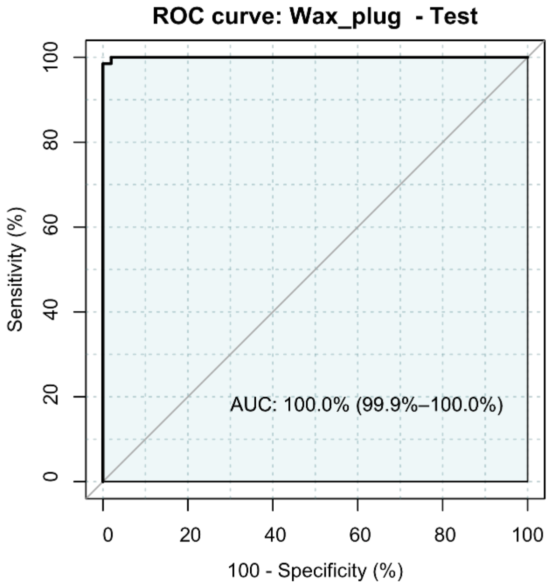

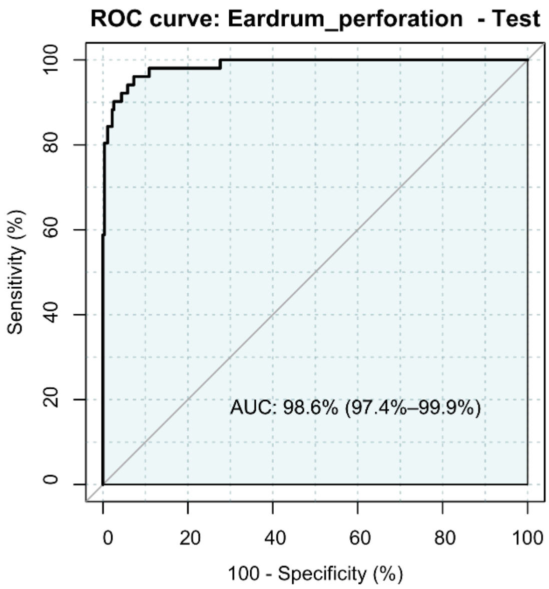
ROC curve: Eardrum_perforation - Test
Sensitivity (%)
100 - Specificity (%)
AUC: 98.6% (97.4%–99.9%)

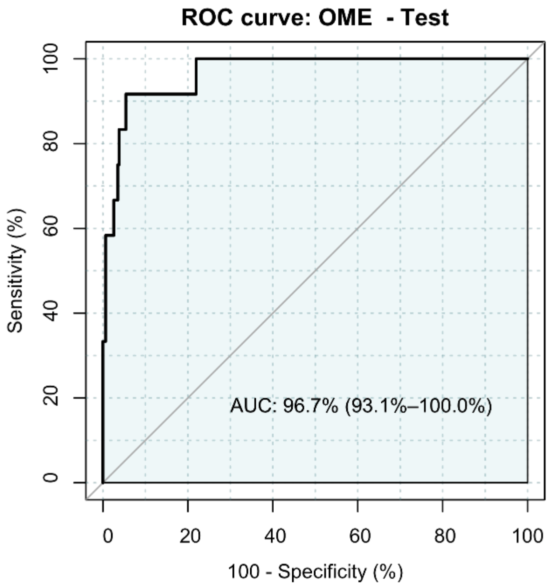
ROC curve: OME - Test
Sensitivity (%)
100 - Specificity (%)
AUC: 96.7% (93.1%–100.0%)

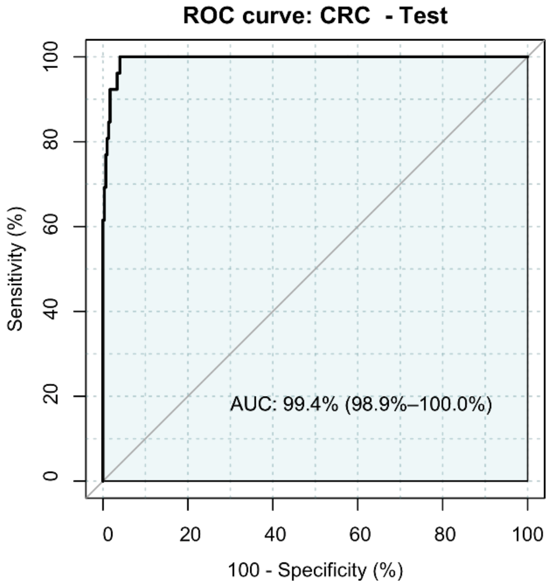
ROC curve: CRC - Test
Sensitivity (%)
100 - Specificity (%)
AUC: 99.4% (98.9%–100.0%)

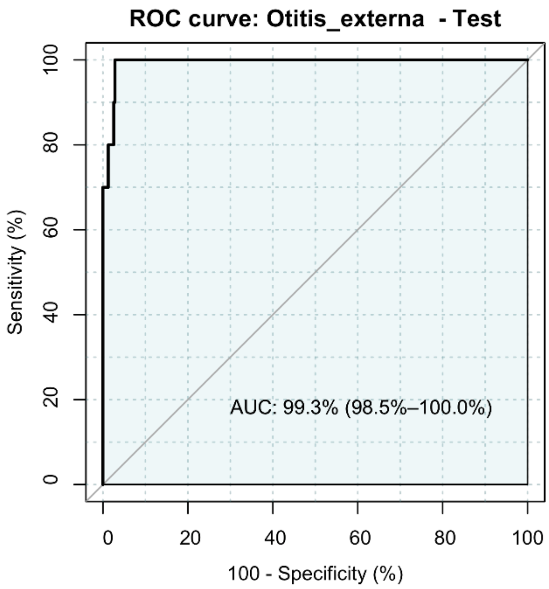
ROC curve: Otitis_externa - Test
Sensitivity (%)
100 - Specificity (%)
AUC: 99.3% (98.5%–100.0%)
0
20
40
60
80
100

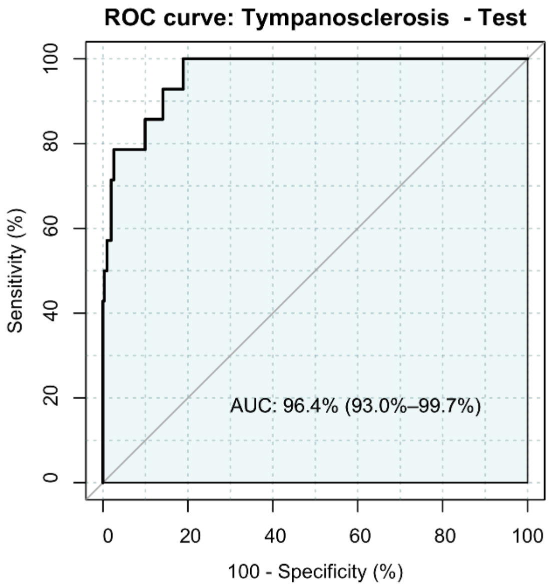
ROC curve: Tympanosclerosis - Test
Sensitivity (%)
100 - Specificity (%)
AUC: 96.4% (93.0%–99.7%)
0
20
40
60
80
100

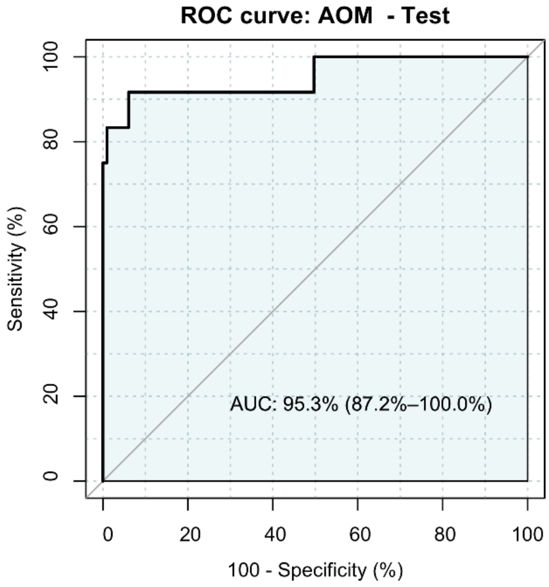
ROC curve: AOM - Test
Sensitivity (%)
100 - Specificity (%)
AUC: 95.3% (87.2%–100.0%)
0
20
40
60
80
100

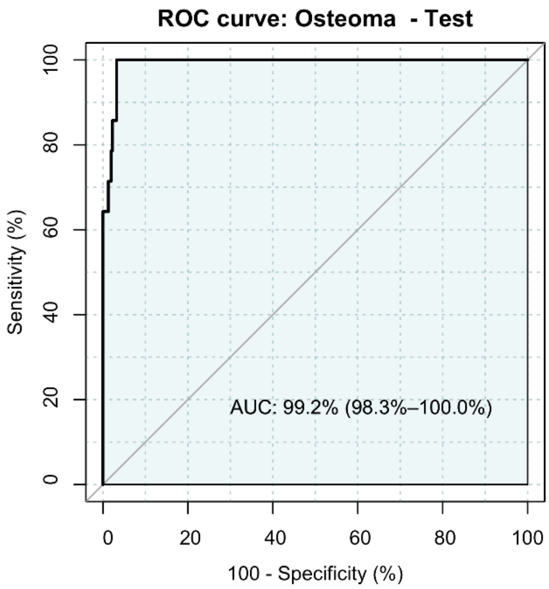

ROC curve: Osteoma - Test
AUC: 99.2% (98.3%–100.0%)
Sensitivity (%)
100 - Specificity (%)

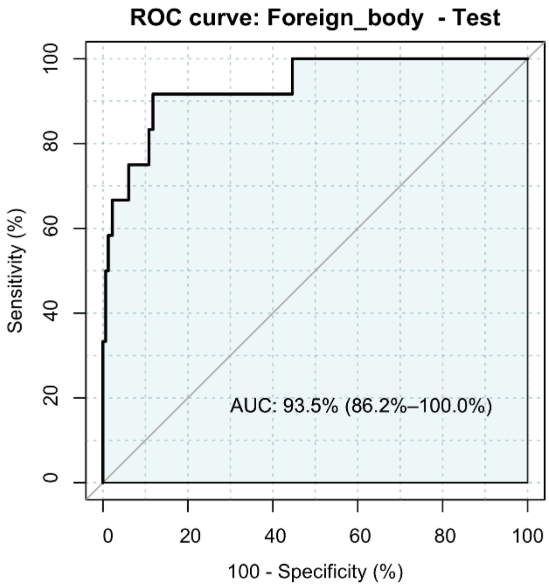

ROC curve: Foreign_body - Test
AUC: 93.5% (86.2%–100.0%)
Sensitivity (%)
100 - Specificity (%)

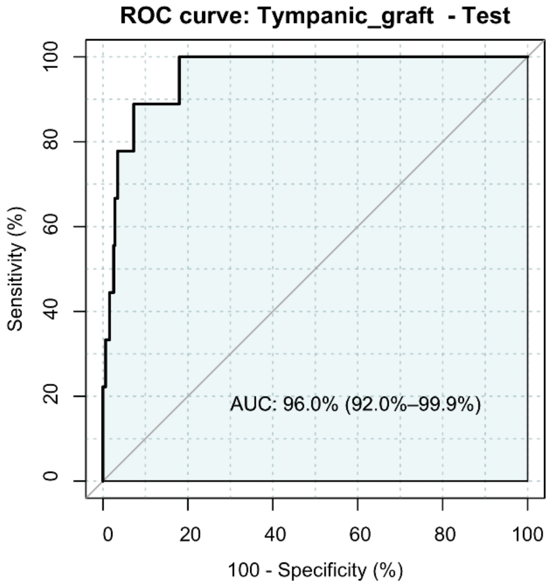

ROC curve: Tympanic_graft - Test
AUC: 96.0% (92.0%–99.9%)
Sensitivity (%)
100 - Specificity (%)
