# Supplementary material for: Development and validation of a smartphone-based deep-learning-enabled system to detect middle-ear conditions in otoscopic images
Source: NPJ Digit Med. 2024 Jun 20;7:162. doi: 10.1038/s41746-024-01159-9 (PMC11189910; doi:10.1038/s41746-024-01159-9)
Supplement: Supplementary file 2 — Reporting summary [file 41746_2024_1159_MOESM2_ESM.pdf]

nature portfolio

Corresponding author(s): Jérémie F. Cohen

Last updated by author(s): May 27, 2024

Reporting Summary

Nature Portfolio wishes to improve the reproducibility of the work that we publish. This form provides structure for consistency and transparency in reporting. For further information on Nature Portfolio policies, see our Editorial Policies and the Editorial Policy Checklist.

Statistics

For all statistical analyses, confirm that the following items are present in the figure legend, table legend, main text, or Methods section.

n/a	Confirmed	
☐	☒	The exact sample size (*n*) for each experimental group/condition, given as a discrete number and unit of measurement
☐	☒	A statement on whether measurements were taken from distinct samples or whether the same sample was measured repeatedly
☐	☒	The statistical test(s) used AND whether they are one- or two-sided<br>*Only common tests should be described solely by name; describe more complex techniques in the Methods section.*
☐	☒	A description of all covariates tested
☐	☒	A description of any assumptions or corrections, such as tests of normality and adjustment for multiple comparisons
☐	☒	A full description of the statistical parameters including central tendency (e.g. means) or other basic estimates (e.g. regression coefficient) AND variation (e.g. standard deviation) or associated estimates of uncertainty (e.g. confidence intervals)
☒	☐	For null hypothesis testing, the test statistic (e.g. *F*, *t*, *r*) with confidence intervals, effect sizes, degrees of freedom and *P* value noted<br>*Give P values as exact values whenever suitable.*
☒	☐	For Bayesian analysis, information on the choice of priors and Markov chain Monte Carlo settings
☒	☐	For hierarchical and complex designs, identification of the appropriate level for tests and full reporting of outcomes
☐	☒	Estimates of effect sizes (e.g. Cohen's *d*, Pearson's *r*), indicating how they were calculated

Our web collection on statistics for biologists contains articles on many of the points above.

Software and code

Policy information about availability of computer code

Data collection	No specific software was used to collect the data.
Data analysis	Baseline Inception-v2 architecture can be accessed on the timm repository (huggingface.co/timm). Further code is available upon reasonable request to the authors.<br>Statistical analysis was done on RStudio, Version 2023.12.1+402.<br>Deep learning modeling was implemented in Python by using TensorFlow libraries.

For manuscripts utilizing custom algorithms or software that are central to the research but not yet described in published literature, software must be made available to editors and reviewers. We strongly encourage code deposition in a community repository (e.g. GitHub). See the Nature Portfolio guidelines for submitting code & software for further information.

Data

Policy information about availability of data

All manuscripts must include a data availability statement. This statement should provide the following information, where applicable:

- Accession codes, unique identifiers, or web links for publicly available datasets
- A description of any restrictions on data availability
- For clinical datasets or third party data, please ensure that the statement adheres to our policy

A sub-sample of the training set, with 100 images per diagnostic class is available upon reasonable request to the authors.

Research involving human participants, their data, or biological material

Policy information about studies with human participants or human data. See also policy information about sex, gender (identity/presentation), and sexual orientation and race, ethnicity and racism.

Reporting on sex and gender	Data on sex and gender were not collected in this study, which relied solely on image analysis.
Reporting on race, ethnicity, or other socially relevant groupings	Race, ethnicity, and other socially relevant groupings were not used in this study, which relied solely on image analysis.
Population characteristics	Population characteristics were neither collected nor analyzed in this study, which relied solely on image analysis.
Recruitment	We recruited all consecutive patients over 5 years old who presented to one private ENT practice in Strasbourg, France, from May 2013 to December 2017. For further performance assessment, a 'held-out' test set was built with unique images collected in the same conditions over 2018-2020, without any overlap with the training and validation sets, ensuring that images from the same ear or different ear from the same person were not included across the validation and test datasets. All participants were evaluated by a single ENT specialist (L.S.) with more than 20 years of clinical experience. For each patient, both ears underwent otoscopic examination.
Ethics oversight	Images (and videos) were taken as part of routine care with oral information. Informed written consent was waived by the Institutional Review Board ('Comité d'éthique de la recherche AP-HP Centre', IRB registration No. 00011928) because of the retrospective nature of the analysis, total deidentification of clinical images, and absence of any other patient information being collected.

Note that full information on the approval of the study protocol must also be provided in the manuscript.

Field-specific reporting

Please select the one below that is the best fit for your research. If you are not sure, read the appropriate sections before making your selection.

☒ Life sciences ☐ Behavioural & social sciences ☐ Ecological, evolutionary & environmental sciences

For a reference copy of the document with all sections, see nature.com/documents/nr-reporting-summary-flat.pdf

Life sciences study design

All studies must disclose on these points even when the disclosure is negative.

Sample size	No formal sample size calculation was performed. The initial dataset was split into training and validation sets.
Data exclusions	Images were excluded if they were out-of-focus, too dark, or blurry.
Replication	Validation was performed on an external test dataset.
Randomization	Not applicable. All otoscopic images included were labeled by the expert ENT specialist and evaluated by the DL system.
Blinding	Not applicable. No blinding was necessary in this study.

Reporting for specific materials, systems and methods

We require information from authors about some types of materials, experimental systems and methods used in many studies. Here, indicate whether each material, system or method listed is relevant to your study. If you are not sure if a list item applies to your research, read the appropriate section before selecting a response.

Materials & experimental systems

n/a	Involved in the study	
☒	☐	Antibodies
☒	☐	Eukaryotic cell lines
☒	☐	Palaeontology and archaeology
☒	☐	Animals and other organisms
☐	☒	Clinical data
☒	☐	Dual use research of concern
☒	☐	Plants

Methods

n/a	Involved in the study	
☒	☐	ChIP-seq
☒	☐	Flow cytometry
☒	☐	MRI-based neuroimaging

Clinical data

Policy information about clinical studies

All manuscripts should comply with the ICMJE guidelines for publication of clinical research and a completed CONSORT checklist must be included with all submissions.

Clinical trial registration	This observational study was not registered in a clinical trial registry because of the retrospective nature of the analysis, total deidentification of clinical images, and absence of any other patient information being collected.
Study protocol	The study protocol was written in French for examination by the Ethics Committee and can be accessed upon request to the authors.
Data collection	We recruited all consecutive patients over 5 years old who presented to one private ENT practice in Strasbourg, France, from May 2013 to December 2017. For further performance assessment, a 'held-out' test set was built with unique images collected in the same conditions over 2018-2020, without any overlap with the training and validation sets, ensuring that images from the same ear or different ear from the same person were not included across the validation and test datasets. All participants were evaluated by a single ENT specialist (L.S.) with more than 20 years of clinical experience. For each patient, both ears underwent otoscopic examination.
Outcomes	Primary outcomes were class-specific measures of diagnostic accuracy (i.e., sensitivity, specificity, and area under the ROC curve).

Plants

Seed stocks	Not applicable.
Novel plant genotypes	Not applicable.
Authentication	Not applicable.
